# The impact of decentralisation on health systems: a systematic review of reviews

**DOI:** 10.1136/bmjgh-2023-013317

**Published:** 2023-12-22

**Authors:** Sujata Sapkota, Amshu Dhakal, Simon Rushton, Edwin van Teijlingen, Sujan B Marahatta, Julie Balen, Andrew CK Lee

**Affiliations:** 1 Manmohan Memorial Institute of Health Sciences, Kathmandu, Nepal; 2 The University of Sheffield, Sheffield, UK; 3 Bournemouth University, Poole, UK; 4 Nepal Open University, Lalitpur, Nepal; 5 Canterbury Christ Church University, Canterbury, UK

**Keywords:** Health systems, Systematic review

## Abstract

**Background:**

Decentralisation is a common mechanism for health system reform; yet, evidence of how it impacts health systems remains fragmented. Despite published findings from primary and secondary research illustrating range of impacts, a comprehensive and clear understanding is currently lacking. This review synthesised the existing evidence to assess how decentralisation (by devolution) impacts each of the six WHO building blocks, and the health system.

**Method:**

We systematically searched five electronic databases for reviews exploring impact of decentralisation on health systems, globally. Reviews, both systematic and non-systematic, published in the English language from January 1990 to February 2022 were included. Data were synthesised across each of six building blocks. Quality assessment of the reviews was conducted using Critical Appraisal Skills Program for systematic and Scale for Assessment of Narrative Review Articles for non-systematic reviews.

**Results:**

Nine reviews, each addressing somewhat different questions, contexts and issues, were included. A range of positive and negative impacts of decentralisation on health system building blocks were identified; yet, overall, the impacts were more negative. Although inconclusive, evidence suggested that the impacts on leadership and governance and financing components in particular shape the impact on overall health system. Assessment of how the impact on building blocks translates to the broader impact on health systems is challenged by the dynamic complexities related to contexts, process and the health system itself.

**Conclusions:**

Decentralisation, even if well intentioned, can have unintended consequences. Despite the difficulty of reaching universally applicable conclusions about the pros and cons of decentralisation, this review highlights some of the common potential issues to consider in advance.

**PROSPERO registration number:**

CRD42022302013.

**WHAT IS ALREADY KNOWN ON THIS TOPIC**

- Scattered information on the impact of decentralisation on the six WHO health system building blocks.

**WHAT THIS STUDY ADDS**

- First study to collate multiple reviews and synthesise their evidence on the impact of decentralisation on the six WHO health system building blocks.
- Reported impacts on the health system building blocks are more negative than positive.
- There is insufficient research in particular on how decentralisation impacts the medical products and technologies and health information system components.
- (Pre-)existing (political and non-political) culture and the financial/economic situation of (sub)national jurisdictions have a strong effect in shaping whether impacts of decentralisation will be positive or negative for the health system and its components.
- There is a lack of research on how decentralisation’s impact on the health system develops over time.

**HOW THIS STUDY MIGHT AFFECT RESEARCH, PRACTICE OR POLICY**

- Decentralisation has the potential to affect the health system in negative ways. Any country considering or adopting decentralisation as a health system reform strategy must be vigilant of the possible negative impacts and design mechanisms to address them.
- More research is needed to understand how health information systems are impacted by decentralisation: a negative impact on health information systems could also undermine impact/outcome analysis.
- Need for longitudinal analysis to better understand if and how the impacts of decentralisation develop over time.

## Introduction

Globally, the decentralisation of health systems is a common health sector reform process that aims to improve health system performance and health outcomes through transferring authority and power from a central (higher) level to peripheral (lower) levels, closer to health service users.^1–3^ This can be accomplished in different ways, including through the regionalisation of healthcare delivery, establishing mechanisms to delegate authority to healthcare institutions at the local/community level, and/or power transfer from higher to lower levels of management/government.^1^

Decentralisation is generally implemented on account of its purported benefits, such as improved and more responsive local decision-making and stronger engagement with local communities.^2 4–6^ However, evidence regarding the extent of such benefits, as well as any negative effects, remains unclear. The impacts of health system decentralisation have been inconsistently reported, with some studies being far more positive than others.^4 7^ Studies suggest that some countries have seen significant positive progress following health system decentralisation; in other cases, a decentralised health system has subsequently been recentralised as a result of the perceived failure of the reform.^4^ Yet, mixed evidence may not only reflect different country experiences: the nature and approach of previous studies also vary greatly, with differences in the type(s)/form(s) of decentralisation studied, methodologies employed to assess the impact and in the health system concepts/components covered.

Health system reforms are the result of policy decisions that can be driven by various motives—not all of them directly related to the health sector itself. In some cases, decentralisation may be specifically focused on attempting to address perceived deficiencies (or bring efficiencies) in the health system. In others, it can result from wider processes of political reform; for example, the implementation of a devolved system of government.^1 2 4 5^

The commonly used WHO health system framework provides a useful lens through which to consider the impact of decentralisation.^8 9^ The framework divides the health system into six components: leadership and governance, health service delivery, human resources for health (HRH), medical products and technologies, health financing and health information systems (HIS). Importantly, the framework emphasises the co-dependencies between these building blocks, with each influencing the other. Thus, while health system decentralisation which involves the transfer of authority and power most obviously relates to the ‘leadership and governance’ building block, we would expect decentralisation (or any other major health system reform) to have consequences across all six. However, the effects may not be uniform across building blocks: indeed, decentralisation could conceivably have positive impacts on some building blocks, and more negative impacts on others.

This review brings together findings from published reviews that have studied the impacts of health system decentralisation that has occurred as a result of the devolution of power/authority from central to lower levels of government. The aim is to understand how decentralisation by devolution impacts each of the six WHO building blocks, and in turn the balance of positive and negative impacts on health systems as a whole. More specifically, the review explores (a) what impacts decentralisation has on each building block, and (b) if (and how) impacts on individual building blocks can lead to consequences for the overall health system. In doing so, the study seeks to contribute to knowledge on the issues that countries considering or embarking on health system decentralisation may face.

## Methods

We used a systematic scoping review methodology to describe existing evidence in the form of published reviews, drawing on methods described by Arksey and O’Malley.^10^ This included ‘systematically searching, selecting and synthesising existing knowledge’^10^ in order to explore and scope available evidence and gaps in knowledge related to the impact of decentralisation on health systems. The review informed a broader programme of research in that area.^11 12^

### Study identification and retrieval

Reviews, both systematic and non-systematic, on the impact of decentralisation on health systems, globally, were searched for in five databases: PubMed, EMBASE, Scopus, SciELO and the Cochrane Library. EMBASE and Scopus were accessed through HINARI.

Search criteria employed broad terms to represent two key concepts: (1) decentralisation and (2) health systems. We limited the search criteria to reviews published in the English language between January 1990 and February 2022 (inclusive), corresponding to the period when studies relating to decentralisation began to be published until the date when the search was conducted. Search terms were adapted as appropriate for each database. The search terms centred around ‘decentralisation’ (eg, decentralisation, devolution, federalisation) AND ‘health and health system’ (eg, healthcare, health governance, health management, health sector, health services, healthcare quality and health planning). Search criteria concepts and the search strategy used for EMBASE are presented as online supplemental material 1.

10.1136/bmjgh-2023-013317.supp1 Supplementary data

Additionally, we searched reviews and grey literature in Google Scholar, PROSPERO, www.greyliterature.com and WHO websites, and hand-searched the reference list of the reviews included and other relevant publications^13 14^ to find additional studies. For articles that could not be retrieved online, corresponding authors were contacted to request a copy via email.

Articles were selected based on predetermined criteria for inclusion and exclusion, as follows:

#### Inclusion criteria

- (Peer-reviewed) reviews (systematic, narrative or scoping) of research articles/empirical studies on the impacts of decentralisation (by devolution) on health systems.

#### Exclusion criteria

- Policy reviews, programme reviews and data reviews (ie, not reviews of original research).
- Document reviews (as primary method).
- Clinical studies.
- Perspectives, opinion pieces, viewpoints or commentaries.
- Reviews published as books.
- Reviews focusing on alternative forms of ‘decentralisation’ (eg, ‘decentralisation’ or delegation of tasks at an organisational/professional level; ‘decentralisation’ from one mode of health service delivery to another—for example, moving patients from acute to home care services).
- Reviews where decentralisation was an outcome/argument, not a study parameter.
- Reviews focusing on policies/programmes of a specific level of government without considering the impact of decentralisation on the system as a whole.

### Study selection and quality appraisal

Studies retrieved from each database were first imported to Zotero reference management software. The studies were screened for duplicates, followed by sequential filtering by titles and abstracts. Articles for full-text review were then selected. SS and AD independently screened articles retrieved from the databases, at the first step, based on titles and abstract, and then full-text review. SS and AD compared and agreed on the final set of studies. Where agreement could not be reached, EvT and SR reviewed the articles and a final decision was agreed.

Quality appraisal of the included studies was conducted using the Critical Appraisal Skills Program (CASP) checklist for systematic reviews^15^ and the Scale for Assessment of Narrative Review Articles (SANRA)^16^ for non-systematic reviews. SS and AD independently assessed the risk of bias and quality of the review. Disagreements were resolved by discussion between authors.

Following assessment, we scored CASP checklist items (yes=2; can’t tell=1; no=0) and categorised systematic reviews with scores 16–20 as ‘high’, 11–15 as ‘medium’ and 0–10 as ‘low’ quality. Non-systematic reviews using the SANRA checklist, as indicated, were categorised as high, medium and low for scores 9–12, 5–8 and 0–4, respectively.

To avoid the potential for bias that may arise from low-quality studies, a decision was made to include only high and medium-quality studies.

### Data extraction and analysis

We extracted general information (such as: authors/publication date, review type, review objectives, context covered by the reviews, health system aspect focused on and quality appraisal of studies included in the reviews) and findings on decentralisation’s impact on the health system (in general and specific to the WHO’s six health system building blocks). Irrespective of the stated objectives/focus, we searched each paper for statements on specific health system building blocks and classified information into relevant health system building blocks as appropriate. Any reference/account of information on building block components presented, even the accounts derived from the studies included in the review, were carefully extracted and collectively analysed. Data extraction used predesigned data extraction forms.

We conducted a collective analysis of the evidence presented by the different reviews and have presented (in the Results section) a narrative synthesis (account) of evidence on decentralisation’s impact on each specific building block, as well as on health systems in general.

The review protocol has been registered in PROSPERO (CRD42022302013) (available at: https://www.crd.york.ac.uk/prospero/display_record.php?RecordID=302013). We report the review as per the Preferred Reporting Items for Systematic Reviews and Meta-Analyses reporting guidelines^17^; the checklist is provided as online supplemental material 2.

10.1136/bmjgh-2023-013317.supp2 Supplementary data

## Results

### Search results

**Figure 1:**
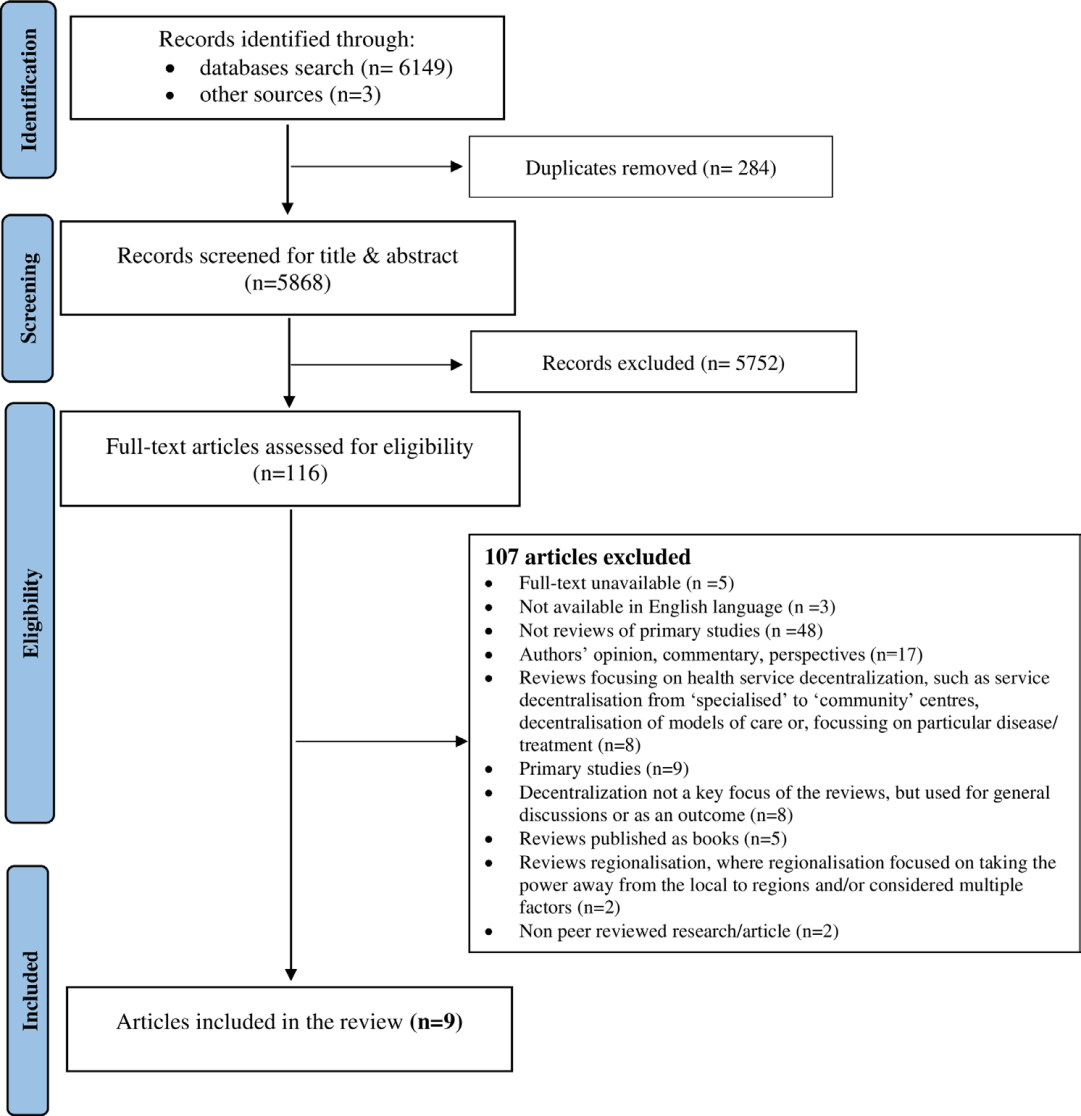
Preferred Reporting Items for Systematic Reviews and Meta-Analyses flow chart of study selection process.

The database search yielded over 6000 articles, and a further three records were identified from searches conducted in Google Scholar and PROSPERO. Following title and abstract screening, 116 articles were considered for a full-text review, and 9^18–26^ were included (figure 1).

### Review characteristics and quality

Of the nine studies included, six were systematic reviews. The number of primary articles included in these reviews ranged from 9 to 76 per review. The reviews covered different geographical contexts and settings; three reviews considered the global scenario^18 25 26^ and two focused on low-income and middle-income countries.^19 23^ A further four reviews focused on more specific regions and countries, including: sub-Saharan Africa,^24^ Indonesia,^22^ Kenya^21^ and Brazil.^20^ Of the three ‘global’ reviews, one narrowed its findings to propose a framework specific to India.^18^

All reviews explored decentralisation’s impact on the health system; however, their focus varied. For example, one explored *experiences*,^24^ another explored *progress and challenges* 21 of the devolved health system. Similarly, health/health system-related issues explored in the included reviews varied: three focused on equity,^21 25 26^ one each on health system performance^18^ and health system inputs, performance and outputs.^23^ Two focused on assessing impacts based on the six WHO health system building blocks.^19 22^ Though one additional review stated that it was ‘*based on application of the WHO’s health system building blocks framework to the principles of management*’, the findings were not categorised according to the building blocks.^18^ A further two studies focused more on the health service delivery component: one looked at human visceral leishmaniasis (HVL) control^20^ and the other at service reform at the local level before and after decentralisation.^24^ A realist review shed light on the importance of contexts and mechanisms of decentralisation in understanding the outcomes/impact.^26^ Finally, although the reviews included studies conducted at different times and intervals following devolution, none specifically analysed how changes developed over time, through a longitudinal analysis.

Five of the six systematic reviews and the realist review conducted quality appraisal of the included studies using standard tools; one systematic review simply mentioned ‘systematic evaluation of quality’, and four reported on the outcome of quality appraisal, highlighting generally low-to-moderate quality of evidence (table 1).

**Table 1:** Review characteristics and quality assessment

Study	Review type	Review objectives (summarised from each review)	Contexts covered (number of articles reviewed)	Health-related aspect(s) focused upon	Quality appraisal (yes/no)	Comment on quality of studies included	Review quality (CASP/SANRA)
Abimbola *et al* 26	Realist review	Understand how relationships between context and mechanism influence the effects of decentralisation.	Global: 25 countries represented (n=51)	Health system equity, efficiency and resilience	Yes	Not reported	High
Dwicaksono and Fox^23^	Systematic review	Identify whether decentralisation processes have impacted health systems.	LMICs (n=16)	Health system inputs, system performance and health outcomes	Yes	Only 10 studies showed relatively low risks of bias	High
Masaba *et al* 21	Systematic review	Systematically review progress and challenges of the devolved healthcare system in Kenya.	Kenya (n=23)	(Progresses and challenges of devolved) healthcare system	Yes	Not reported	Medium
Menon *et al* 20	Literature review	Highlight shortcomings of HVL control programme in decentralised context; identify research gaps for HVL control/prevention.	Brazil (not reported)	Health system delivery/effectiveness (HVL control)	No	Not applicable	High
Cobos Muñoz *et al* 19	Systematic review	Provide an updated and comprehensive assessment of the effects of decentralisation in LMICs.	LMICs: 26 countries represented (n=54)	WHO health system building blocks	Yes	28 quantitative studies, 16 moderate quality and remaining weak. 11 qualitative: only one complied with CASP criteria. 11 studies complied with 75+% of criteria, 7 with 50–75% of criteria and 8 failed >50%.	High
Panda and Thakur^18^	(Focused) review	Examine dimensions and determinants of health system performance; methodological challenges in dealing with performance measurement; and propose derivatives in the form of a conceptual framework that is holistic in approach and specific to India.	Global: narrowed to propose conceptual framework holistic in approach but specific to Indian context (n=76)	Dimensions (definitions, functions and instruments; efficiency; quality; health outcomes; conceptual approaches; measuring performance; measurement tools); determinants (health facilities; agents of local decision-making; end-users) of health system performance	No	Not applicable	Medium
Rakmawati *et al* 22	Systematic review	Identify post‐decentralisation problems in Indonesia’s health system at district level based on WHO building blocks; their points of articulation, priorities for policy formulation, adequate intervention, evaluation. Propose policy recommendations for district health system’s performance.	Indonesia (n=29)	WHO health system building blocks	Yes	Quality of the studies varied. Because of the nature of decentralisation, no RCTs, resulting in a relatively low strength of evidence	Medium
Sumah *et al* 25	Systematic review	Assess empirical evidence on implications of health system decentralisation on equity in health, healthcare and health financing.	Global: 6 countries represented (n=9)	Equity of healthcare access or utilisation, equity in health status/outcome, financing healthcare	Yes	Quality scores generally above average. 4 studies high quality; others rated moderate quality.	High
Zon *et al* 24	Systematic review	Review experiences of local governments providing local health services during and after decentralisation reforms.	Sub-Saharan Africa (n=21)	Broadly, health service delivery and healthcare	Conducted ‘systematic evaluation’	Not reported	Medium

CASP, Critical Appraisal Skills Program; HVL, human visceral leishmaniasis; LMICs, low-income and middle-income countries; RCTs, randomised controlled trials; SANRA, Scale for Assessment of Narrative Review Articles.

Of the nine reviews included, we categorised five as ‘high’ quality and the remaining four as ‘medium’ quality (table 1).

### Evidence on impact

#### Impact of decentralisation on individual health system building blocks

Between them, the nine reviews included in this review addressed all six health system building blocks, although not to the same extent. Information regarding health service delivery could be derived from all studies^18–26^; leadership and governance^18–24 26^ and health financing^18 19 21–26^ from eight studies; and human resources from seven studies.^18–22 24 26^ Fewer studies, six and four, respectively, provided information on medical products and technologies^19–22 24 26^ and HIS^19–22^ (table 2). The reported impacts for each building block varied across studies. Although the pros and cons of decentralisation were mixed across building blocks, overall, more negative than positive impacts were reported for most health system components.

**Table 2:** WHO health system building blocks addressed in the included reviews

Study	WHO health system building blocks					
	Leadership and governance	Human resources for health	Health service delivery	Health financing	Medical products and technologies	Health information system
Abimbola *et al* 26	✔	✔	✔	✔	✔	–
Dwicaksono and Fox^23^	✔	–	✔	✔	–	–
Masaba *et al* 21	✔	✔	✔	✔	✔	✔
Menon *et al* 20	✔	✔	✔	–	✔	✔
Cobos Muñoz *et al* 19	✔	✔	✔	✔	✔	✔
Panda and Thakur^18^	✔	✔	✔	✔	–	–
Rakmawati *et al* 22	✔	✔	✔	✔	✔	✔
Sumah *et al* 25	–	–	✔	✔	–	–
Zon *et al* 24	✔	✔	✔	✔	✔	–

##### Impact on leadership and governance

Reviews indicated increased opportunities for corruption following decentralisation. Although one review included a study where significant impact of decentralisation on corruption was not found,^23^ others reported political interference in health personnel recruitment processes,^24^ local authorities’ interference in decision-making,^19^ political harassment of civil servants, increased nepotism,^18 24^ and low transparency in budgeting and expenditure^22^ as key challenges following decentralisation. Furthermore, bureaucratic response, for example, resistance of civil servants to power structure changes, difficulty of persuading staff and their families to accept posts in peripheral areas, and increased local-level authority, fuelled opportunities for patronage and corruption.^18^

At the same time, the reviews showed that decentralisation does not necessarily widen the decision space for local jurisdictions (or for the health sector) in practice, and the extent to which subnational jurisdictions can make decisions, and influence health/health systems, varies. Neither does effective devolution of decision-making necessarily lead to positive impacts: instead, it could limit the benefits from economies of scale and resource coordination across local units.^26^ Limited autonomy or choice, typically for human resource and financing functions, was found.^24^ Furthermore, decentralisation was reported to dilute hierarchical relationships, but multiply mutual accountability relations between different levels of government.^26^ While the impact of diluted hierarchy can be negative or positive, the increased mutual accountability relations were reported to enhance ‘back-up mechanisms’ with the potential for one government level to address a need that another government level cannot. However, gaps between/variation in (constitutional) expectations versus (on the ground) reality^26^ meant that these potential benefits did not always accrue in practice. Coordination problems between central level and local authorities reportedly resulted in priority programmes being blocked.^19^

**Figure 2:**
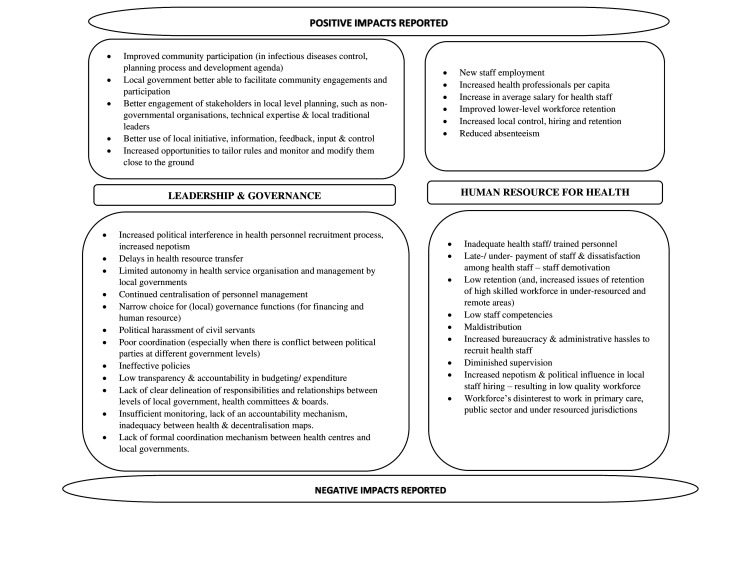
Positive and negative impacts reported for leadership and governance and human resources for health components.

A consistent positive impact reported across studies was the influence of decentralisation on community participation—the prospects for which increased with localised decision-making for health services.^19 21 24 26^ Zon *et al* reported local units were very useful in facilitating community participation in infectious disease control, in the planning process and in improving healthcare quality.^24^ More direct and improved participation of the local community in health development agenda and health services was also reported.^19 21 26^ Information exchange between local bodies, such as non-governmental organisations and traditional leaders, reportedly flourished in a decentralised context^26^ (figure 2).

##### Impact on human resource for health

The reported impact of decentralisation on HRH was inconsistent across studies. Overall, the impacts reported were more negative than positive. Positive impacts included improved prospects of capacity building, supervision and scrutiny of primary health services, reduced absenteeism, increased availability of funds for HRH management at subnational levels and improved planning in health workforce recruitment due to effective utilisation of local information.^24^ Additionally, a study indicated improved prospects of low-skilled workforce retention that could be resourced locally,^26^ and another documented an increase in health staff numbers per capita after decentralisation.^19^ However, most other reviews reported a decrease in health staff at subnational/local levels post-devolution.^20–22 24^ Negative impacts on HRH additionally included maldistribution,^19 22^ low retention/shortage,^19 20 22^ delayed (and under)payment,^18 22^ low workforce competencies,^22 24 26^ recruitment and retention challenges in the case of highly skilled health workers,^24 26^ and health workers’ preference to work in secondary care and in richer (and more highly resourced) jurisdictions and/or in the private sector.^18 22 26^

Human resource management was seen as a challenge for local-level and for lower-resourced jurisdictions. Human resource recruitment, retention/transfer, training and promotion were impacted by nepotism following decentralisation, particularly when bureaucratic workload and diminished supervision from a higher level led to a lack of accountability in human resource management processes.^19 26^ Additionally, pre-existing human resource inequities and workforce retention issues, especially in the case of highly skilled staff, in rural/remote areas, could worsen post-decentralisation, with wealthier, urban jurisdictions having an advantage because of their ability to fund attractive remuneration packages and attract (higher) skilled workers^26^ (figure 2).

##### Impact on health service delivery

All nine reviews provided some information on the health service delivery component, making it the most widely researched building block. Health service delivery impact assessment involved analysis of a range of outcome variables, including health outcomes, immunisation coverage, mortality, access to health services, and detection and management of diseases. The impacts reported for these variables were mixed and inconsistent across reviews and between studies included in the reviews. For example, while immunisation coverage post-decentralisation was reported to be ‘highly variable between districts’ by one study,^22^ another reported a decline in Expanded Program on Immunization coverage following decentralisation.^24^ Panda and Thakur reported a study where differences in immunisation post-decentralisation were found between low-income and high-income countries and noted that child immunisation schemes performed better in low-income countries.^18^ While the commonly theorised advantage of services getting closer to users (improved access) post-decentralisation was noted to be true for some countries,^25^ inequity in access to maternal health services was also reported.^22^ Positive impacts post-decentralisation were reported for curative services and hospital attendance,^24^ access^19^ and (medical and non-medical) infrastructure development.^21^ Infrastructure developments following devolution were reported to aid improvement in health outcomes.^21^

**Figure 3:**
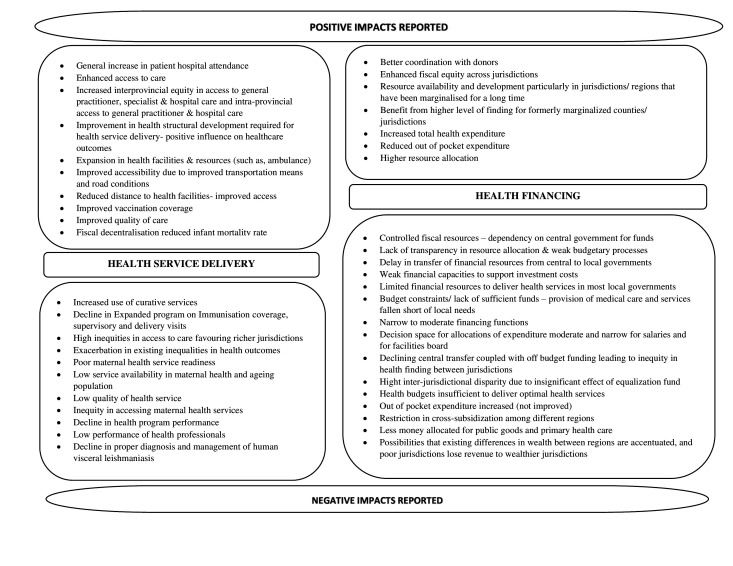
Positive and negative impacts reported for health service delivery and health financing components.

Analyses of the impact on health service delivery parameters/variables are influenced by (pre-)existing contextual issues that impact service utilisation practices. The reviews particularly highlighted the importance of financial/socioeconomic factors and health policies as impact predictors. Two reviews that focused on assessing equity reported that, in general, health service outcomes in decentralised contexts are more favourable for richer (better resourced) jurisdictions.^25 26^ Nonetheless, a study included in one of the reviews noted that fiscal decentralisation was associated with reduced infant mortality in poorer jurisdictions, also highlighting that such outcomes ‘depended greatly on the socioeconomic conditions of the localities’^18^ (figure 3).

##### Impact on health financing

In addition to the amount of funds available/allocated to health, particularly to the subnational jurisdictions, the reviews reported impacts of decentralisation on funding mechanisms, such as control of financial resource allocation, budget transfer processes and how available funds are used. The impacts reported were mixed, and variables used to assess impact varied between reviews and studies included in the reviews.

Positive impacts, such as increases in total health expenditure, decrease in out-of-pocket expenditure and improved resources post-decentralisation, were reported.^19^ A review highlighted that fund allocation by multiple governments in a decentralised context aided the health sector and promoted health infrastructure development.^21^ Better coordination with donors led to increased funding,^24^ and increased funding allocation to long-marginalised local levels^21^ were other advantages reported for the health financing component.

On the other hand, the negative impacts reported included poor funding in the health sector overall following devolution,^21 24^ shifts from public to private goods to generate revenues,^18 26^ restricted cross-subsidisation among different regions,^19 26^ controlled financial resources allocation,^24^ weakness in budgetary procedures,^24^ weak financial capacities and delay in fund transfers from central to local governments.^24^

Fiscal equity was the central theme in a few reviews. Impacts on fiscal equity, or equity in financing mechanisms, were also mixed and context driven. Fiscal equity post-decentralisation was reported to vary, with increased equity in healthcare financing being reported for some countries and reduced equity for others.^25^ Decentralisation can exacerbate inequity in financing mechanisms and was accentuated by pre-existing differences in wealth between regions and where the costs of cross-border movement to access health services are covered by patients’ jurisdiction of residence. Poorer jurisdictions lose revenue while wealthier ones are at an advantage as their capacity to spend on health is better, leading to better institutional capacity, health system performance and health outcomes^26^ (figure 3).

##### Impact on medical products and technologies

Consistent negative impacts of decentralisation on medical products and technologies were reported across studies. The six studies that provided information on this building block collectively offered insights on availability, accessibility, utilisation and price of medical commodities in a decentralised context (or how they changed after decentralisation). Inadequate availability of general and essential^19 21 22 24^ medicines, including antimalarial medicines, blood products and infant vaccines,^22^ and of equipment such as malaria diagnostic tools, HVL control spraying equipment to curb large-scale HVL spread^20^ was reported. Rakmawati *et al* also noted reduced availability of medicines during an influenza pandemic.^22^ Problems with funding and medicine procurement,^24^ increased bureaucracy and lack of management (technical/specialised) skills^19 21^ were the reported reasons for medical commodity shortages. The underutilisation of medical equipment in particular, as Masaba *et al* point out, was a consequence of limited specialised personnel and the rapidly advancing medical equipment/infrastructure trend.^21^ An imbalance/mismatch between assigned responsibilities, resources available and authority granted at the peripheral level post-decentralisation collectively led to less than desired outcomes—effective availability, accessibility and utilisation—for this building block.^19^

**Figure 4:**
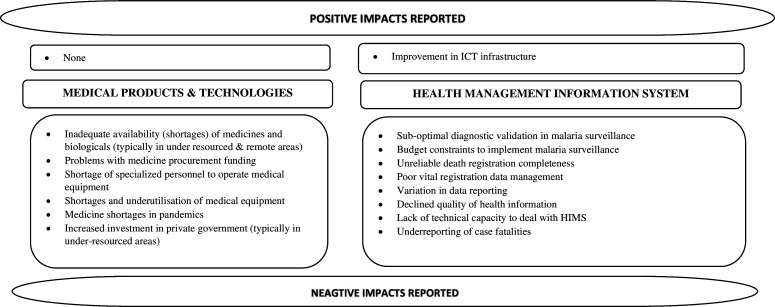
Positive and negative impacts reported for medical products and HIS components.

High variation in medicine prices following decentralisation was noted.^22^ Decentralisation diminished economies of scale.^26^ The lack of centralised supply and procurement raises challenges for poorer and more remote facilities that are under-resourced, and a possible need to resort to other mechanisms such as borrowing medicines across facilities.^21^ Poorer local jurisdictions often invested more in medicines (and curative services) which are seen as ‘revenue-generating commodities’ rather than sustainable preventive promotive health mechanisms^26^—worsening inequity in the longer term (figure 4).

##### Impact on health information systems

Health information systems are the least studied of all WHO health system building blocks in the context of decentralisation. Scarcity of data on this component in relation to decentralisation, and typically of quantitative indicators, was also pointed out by one review.^19^ Of the four reviews that touched on this component,^19–22^ improvement in information and communication technology infrastructure was reported as an example of ‘progress’ post-decentralisation in one study.^21^ All others reported issues with data and information management systems, for example, decline in quality of health information after decentralisation,^19^ data incompleteness, and under-reporting of morbidity and mortality.^20 22^ Lack of technical capacity was a reason given for declining quality in health information following decentralisation^19^ (figure 4).

### Impacts of decentralisation on the health system as a whole

The variability of the impacts of decentralisation on health system building blocks, limitations in the evidence base and the rarity of reviews that covered all six building blocks make it challenging to form a conclusive judgement as to how decentralisation impacts health systems overall. The assessment of whether or not decentralisation is a beneficial health system reform is additionally complicated because of the need for contextualisation: as the reviews show, the effects vary across different parts of the country and between subnational jurisdictions,^22^ as well as between countries. Other challenges included the varied methodologies employed. The reviews, nonetheless, demonstrated that the impacts on leadership and governance and health financing components can lead to broader impacts on the overall health system (see: Impact on leadership and governance and Impact on health financing sections). However, even here context is vital: how these building blocks functioned before devolution is a key influencer in shaping the health system post-devolution.

Where included reviews did attempt to provide holistic judgements of positive or negative impacts on health systems as a whole, they revealed a mixed picture. The reviews found those impact(s) are strongly influenced by pre-existing (political and non-political) contexts. The reviews underscore the importance of closely examining contexts and mechanisms to understand the impacts of decentralisation and to inform implementation strategies.^18 23 25 26^ Benefits of, and a need for, context-specific analysis are also demonstrated by comparatively focused recommendations provided by the reviews looking at specific country contexts and health system issues^20–22^ as compared with studies with broader focus.^19 23^ The reviews provide some evidence that (a) the pre-existing economic situation of localities; (b) the level of jurisdictional autonomy/independence (shifts in decision space); and (c) existing policies can influence decentralisation’s impact.

Aside from the contextual nature of decentralisation, the reviews demonstrated a lack of unanimity on how to measure health system concepts such as efficiency, equity and health system performance.^18^ Operationalisation and assessment mechanisms of these concepts varied across reviews and between studies included in the reviews. For example, one review considered ‘quantifiable’ indicators, such as changes in health service utilisation, service coverage and quality of services as measures for health system performance^23^; Panda and Thakur talked about the dimensions of healthcare performance, although also considering ‘definable, *preferably* measurable and actionable attributes of the system’, however, they also considered broader contextual dimensions, such as environment and culture,^18^ which are not necessarily quantifiable. The same review specifically emphasised the need for taking into account the health system concepts and measurement tools from local-level decision-makers’ perspectives for implementable recommendations and to aid informed decision-making.^18^

Deriving specific conclusions on decentralisation’s impact, and how they result from a balance of the impacts on the individual building blocks, is also challenged by research methodology limitations. Varied and ‘proxy’ health system outcome variables, different ‘assumptions’ and diverse research methods across studies make comparison and synthesis difficult.^18 19^ Research designs and methodological approaches have a direct impact on the strength of the evidence generated; for example, the lack of randomised controlled trials (which are generally seen as unsuitable methods for decentralisation impact assessment) reduces evidence quality. Furthermore, research methods can impact findings. A review reported that almost contradicting evidence was generated by studies employing quantitative and qualitative approaches.^19^ For example, while most quantitative assessments showed positive impacts for the health financing components, qualitative assessments revealed otherwise.^19^

The reviews also highlighted the difficulties involved in identifying causality in terms of whether reported impacts have actually resulted from decentralisation, or are consequences of pre-existing situations (with decentralisation exacerbating or alleviating those longer-term issues). For example, health worker shortages and retention issues, often reported as an impact of decentralisation,^20 22^ are often not *caused* by decentralisation, in many cases, predating it—although they may worsen in a decentralised context, especially in low-resourced localities.^26^

## Discussion

Our analysis shows that decentralisation has wide-ranging impacts across the health system, and these impacts are not uniform across the different building blocks. Decentralisation can have significant negative impacts on the different building blocks, but can also bring benefits in some areas. How the positive and negative impacts of decentralisation aggregate, and their net effect on the health system as a whole, cannot be easily determined. This is in part because of the importance of context, but also as a result of varied methodologies leading to conflicting findings across studies, and the fact that many of the included reviews examine only some of the health system building blocks. Nonetheless, it is clear from the reviews that the impacts on the leadership and governance and health financing components are fundamental in shaping broader consequences for health systems post-devolution.

Of the six WHO health system building blocks, health service delivery was the most frequently addressed building block. The tendency to focus on health service delivery is in many ways understandable because it is the most visible aspect of the health system, and indeed service delivery is the ultimate aim of the system. It is, therefore, an indicator of health system functioning. Meanwhile, the medical products and technologies and HIS building blocks are the least studied, although both are fundamental to smooth health system operations and, ultimately, service quality. The availability of medical products and technologies is closely connected to the quality of service delivery, and ultimately to health outcomes. Apart from being important for supporting decision-making,^27^ information systems are particularly crucial for capturing, analysing and understanding the impacts of decentralisation. With decentralisation being a ‘process’, its impact can change over time.^28^ Longitudinal data are key to understanding the impact of decentralisation over time and the problems commonly identified in the reviews, such as poor data quality or inefficient reporting systems that jeopardise this. Additional studies focusing on these relatively neglected building blocks are needed.

Leadership and governance and health financing emerge as fundamental shapers of health system progress, having a strong influence on the process of transition, and directly affecting the other building blocks.^29 30^ The reported explanations for the positive impacts on the health service delivery component, for example, relate directly to the visibility of service delivery, which tends to be a good motivator of political will as compared with lower profile ‘back office’ functions. Similarly, the reviews show that bureaucratic disruption that often accompanies the devolution of power and responsibilities can have negative consequences across the system.^19^ Whether financial resources increase or decrease post-decentralisation—and how they are distributed across the country—is also fundamental. For example, administrative disruption and financial limitations can both have a significant impact on HRH which is subjected to added political and technical challenges post-devolution.

Devolution, in principle, offers opportunities for local governments/jurisdictions—and even citizens—to seize opportunities to shape the health system to better serve their needs and desires.^2 31^ While this is one of the key purported advantages of decentralisation, lower-level decision-making results in ‘differences’, creating ‘mini’ health systems that vary across jurisdictions—raising the prospects of inequalities in service provision or access. Importantly, the reviews show that decentralisation can exacerbate pre-existing inequalities, and the relative ‘wealth’ of jurisdictions appears to be a relatively strong predictor of decentralisation’s impact. This suggests that poorly resourced areas will need enhanced support if they are to benefit from decentralisation. Maintaining equity/equality^32^ across jurisdictions in a true sense can only be ensured if mechanisms are in place that track and ensure a ‘need-based’ mobilisation of resources.

The health system does not exist in isolation, and the ways in which broader policy and politics develop post-decentralisation have a direct impact on the health sector/system, as do the constitutional arrangements for devolution of authority and resources from central governments to lower levels. Our findings indicate that the impact of decentralisation is shaped by how politicians and bureaucrats respond to the changing relationships between different governance levels. Health objectives may not always be the key driver of devolution of power and authority, instead being secondary to other political agendas. Opportunities and incentives for corruption and political interference, accountability deficits and failures in coordination between different government levels can all exacerbate or be limited by wider issues of political culture. Health systems undergoing decentralisation would benefit from focusing particular thought and planning in these areas—and from careful consideration of the policies that could help address some of the negative changes highlighted in the reviews.

This review highlights the difficulties in generating conclusive evidence of impact (both holistically and on individual building blocks) as both decentralisation and health systems are dynamic and complex to measure, and the mechanisms through which impacts evolve over time are highly contextual: initial bureaucratic disruption, for example, may later give way to a better functioning system.

International comparisons, meanwhile, although critical to improving understanding across different systems and to fostering cross-cultural and cross-political insights, are insufficiently robust to generate concrete implementable or predictive evidence.^3–5 33 34^ Oftentimes, decentralisation mechanisms can be traced back to, or be shaped by, specific countries’ unique evolutionary histories.^35–37^ Culture, contexts, values, motivations—both political and non-political—to devolve, all vary,^5 6 35–38^ and all shape the outcomes.

Furthermore, health systems and performance measurement face multiple ‘technical’ challenges—including a lack of health system assessment standards and indicators, patchy data and inconsistent reporting mechanisms.^39–42^ Precise assessment of the impacts of decentralisation on an entire health system would also need methods capable of capturing (and measuring) the integrated system in a dynamic context—achieving this is severely limited by methodological challenges. In light of the complexities and contextual impacts, tailored assessment methods might have to be developed (for example, using Delphi methods) engaging political and health system experts.

## Conclusion

This review brings together assessments of the impacts of decentralisation conducted in a variety of settings, scope and globally, and suggests that devolution has the potential to create challenges to the health system. Decentralisation, when it occurs as a result of power devolution, can impact all six WHO health system building blocks, but the effects are not uniform. While the impacts on leadership and governance and financing are key influencers of the overall impact, how devolution’s impacts on individual health system building blocks translate to broader health system consequences is difficult to predict. To generate implementable findings, research could focus on discrete aspects of decentralisation and health system concepts taking into account a close consideration of contextual factors and mechanisms in that particular setting. That said, the different impacts reported here, and the causal mechanisms identified, at least provide countries contemplating decentralisation with a list of potential issues to consider in advance.

## Data Availability

All data relevant to the study are included in the article or uploaded as supplemental information.
