# Supplementary material for: The impact of decentralisation on health systems: a systematic review of reviews
Source: BMJ Glob Health. 2023 Dec 22;8(12):e013317. doi: 10.1136/bmjgh-2023-013317 (PMC10749071; doi:10.1136/bmjgh-2023-013317)
Supplement: Supplementary data [file bmjgh-2023-013317supp001.pdf]

Search Strategy Concepts

**Key Concept**	**Key search terms used (each term was combined using OR operator)**
Decentralisation	decentralisation, decentralization, devolution, delegation, deconcentration, federalisation, federalization
AND	
Health and Health System	"Health Care", "Health System", "Health organisation", "Health Governance", "Governance of Health", "Health management", "Health Sector", "Health Services", "Health Care Facilities", "Health Facilities", "Delivery of health care", "Patient Care", "Patient Care Management", "Health Care Quality", "health administration", "health planning", "health commissioning"

Search Strategy employed for EMBASE:

(decentralisation OR 'decentralization'/exp OR decentralization OR devolution OR delegation OR deconcentration OR federalisation OR federalization) AND ('health care'/exp OR 'health care' OR 'health system'/exp OR 'health system' OR 'health organisation'/exp OR 'health organisation' OR 'health governance' OR 'governance of health' OR 'health management'/exp OR 'health management' OR 'health sector' OR 'health services'/exp OR 'health services' OR 'health care facilities' OR 'health facilities'/exp OR 'health facilities' OR 'delivery of health care'/exp OR 'delivery of health care' OR 'patient care'/exp OR 'patient care' OR 'patient care management'/exp OR 'patient care management' OR 'health care quality'/exp OR 'health care quality' OR 'health administration'/exp OR 'health administration' OR 'health planning'/exp OR 'health planning' OR 'health commissioning') AND [1990-2022]/py

Limits applied: Language: English & Article type: Review
